# Identifying Prognostic Factors in Brain Metastasis Patients Using MRI Morphological Features: A Machine Learning and Survival Analysis Approach

**DOI:** 10.3390/diagnostics16071017

**Published:** 2026-03-28

**Authors:** Daniela Pomohaci, Emilia-Adriana Marciuc, Bogdan-Ionuț Dobrovăț, Oriana-Maria Onicescu, Sabina-Ioana Chirica, Costin Chirica, Mihaela-Roxana Popescu, Danisia Haba

**Affiliations:** 1 Doctoral School, Grigore T. Popa University of Medicine and Pharmacy Iasi, 16 Universității Str., 700115 Iasi, Romania; danielapomohaci@yahoo.com (D.P.);; 2 Department of Oral and Maxillofacial Surgery, Faculty of Dental Medicine, Grigore T. Popa University of Medicine and Pharmacy Iasi, 16 Universității Str., 700115 Iasi, Romania; emma.marciuc@gmail.com (E.-A.M.); bogdan.dobrovat@yahoo.com (B.-I.D.); danihaba@yahoo.com (D.H.); 3 Department of Radiology, Emergency Hospital Professor Doctor Nicolae Oblu, 700309 Iasi, Romania; 4 Faculty of Computer Science, “Alexandru Ioan Cuza” University, 700506 Iasi, Romania; oriana.oniciuc@info.uaic.ro

**Keywords:** brain metastasis, overall survival, artificial intelligence, machine learning, MRI data analysis

## Abstract

**Background/Objectives**: Our study aims to identify potential new MRI features of brain metastases (BMs) that could be further used in overall survival (OS) assessment. **Methods**: A total of 109 patients with BMs were included. Kaplan–Meier analysis, the log-rank test, and Cox Regression were implemented in the survival analysis. The first ten significant features were incorporated into four distinct machine learning (ML) algorithms to predict six-month survival. **Results**: Survival analysis revealed that multiple brain lesions and synchronous presentation were associated with a poor prognostic value (HR > 1; *p* = 0.01, *p* = 0.02). Other features demonstrated a protective effect on OS including the absence of extracranial lesions (HR < 1, *p* = 0.04) and the presence of solid enhancement (HR < 1, *p* < 0.05). In this observational cohort, treatment was associated with longer OS—including surgery, gamma knife radiosurgery, whole brain radiation therapy, and chemotherapy—compared to best supportive care (HR < 1, *p* < 0.005); these treatment-related hazard ratios are not interpreted causally. The shallow Neural Networks model was the top-performing ML model, achieving an AUC of 0.93 (CI = 0.89–0.97). According to the Shapley Additive Explanations analysis, the solid enhancement type had a positive impact on OS, whereas a higher number of lesions, larger volumes and a cystic morphology were associated with negative outcomes. **Conclusions**: Our results confirm that including morphological MRI features of BMs in the prediction of OS significantly contributes to the enhancement of ML algorithms’ prediction and discriminatory capacity.

## 1. Introduction

Brain metastases (BMs) are the most frequent malignant brain lesions in adult patients and have a poor prognosis, with an overall survival (OS) ≤ 12 months [1,2,3].

The treatment of secondary lesions depends on various factors determined both by the patient’s characteristics and the imaging features of BMs [4].

Treatment varies from local therapies such as surgery and stereotactic radiosurgery (SRS) or fractionated radiotherapy (SRT) to whole-brain radiotherapy (WBRT), systemic therapies (specific to the primary tumor), or palliative and symptomatic therapies [5].

OS in metastatic brain patients depends on both the intracranial and extracranial containment of the disease [6]. Prognostic factors previously used in the appreciation of OS, are: the performance status of the patient, the age of the patient, the primary tumor, the extent of extracranial disease, the number and size of BMs, their location and the presence of leptomeningeal disease. These factors contribute to the graded prognostic assessment (GPA) score [7,8]. The disease-specific GPA (DS-GPA) is a specific scale where more prognostic elements are taken into consideration depending on the primary tumor [9,10].

The follow-up consists of repeating magnetic resonance imaging (MRI) contrast examinations at 48 h after surgery and at intervals of two–three months [4]. The MRI protocol includes at least pre- and post-contrast T1-weighted (T1W), followed by T2-weighted (T2W) or T2-weighted Fluid Attenuated Inversion Recovery (T2 FLAIR) [11].

Only the dimension and number of the lesions are used as imaging criteria in the response assessment in neuro-oncology (RANO) and response evaluation criteria in solid tumors (RECIST 1.1) follow-up methods [12]. These criteria are implemented in clinical trials to assess BMs’ response to different therapeutic strategies [12].

The application of artificial intelligence (AI) methods in the diagnosis, survival appreciation, and differentiation between recurrence and post-treatment complications are widely explored in brain tumors, with a specific focus on primary tumors rather than secondary lesions [13,14,15].

Both machine learning (ML) and deep learning (DL) models are used, the latter in particular with imaging features such as quantitative data in the context of radiomics [16,17].

AI’s focus in metastatic brain patients is mainly directed towards the differential diagnosis of glioblastoma and evaluation of treatment response, neglecting other areas [17,18,19]. The identification of the primary tumor by the MRI characteristics of the BMs is a possible application of DL in the management of metastatic patients, especially useful when the primary tumor is unknown [20].

In particular, survival analysis is assessed in post-radiotherapy groups of patients, where protocols are standardized [16,17,21]. ML models capable of predicting survival in BM patients remain in the development phase and the majority use primarily clinical data only [6,16].

The study aims to test the hypothesis that the inclusion of morphological MRI data increases ML algorithms’ capability in the prognostic estimation of OS in BM patients. As AI methods and MRI are both continuously evolving, it is safe to speculate that morphological features could soon be used as imaging follow-up criteria in patients with BMs and other brain tumors [22,23]. Contributing to the further use of potential novel imaging characteristics as prognostic factors is another objective, as this could significantly improve clinical decision-making processes [16].

## 2. Materials and Methods

We retrospectively analyzed patients that were diagnosed and treated for BMs in our hospital from 2019 to 2023. Our study has a single-centered observational nature.

### 2.1. Patient Inclusion and Exclusion Criteria

In total 538 cases were analyzed. Patients underwent careful selection following predefined inclusion and exclusion criteria.

**Figure 1:**
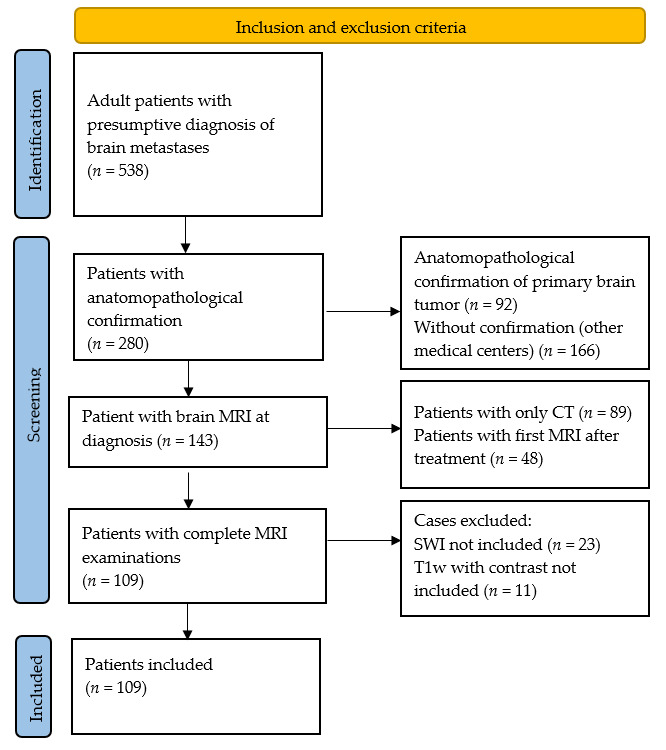
Patient selection process (flow chart from Page MJ et al., BMJ Publishing Group; 2021 [24]).

The Prisma flow chart shows the process of patient selection (Figure 1).

Inclusion criteria are:

1. Adult patients (aged over or equal to 18 y. o.);
2. Histopathological confirmation of brain metastases;
3. At least one initial MRI with contrast, before any treatment;
4. Initial MRI protocols consisting of T1W, T2W, T2 FLAIR, susceptibility-weighted imaging (SWI), diffusion-weighted imaging/apparent diffusion coefficient map (DWI/ADC) and contrast enhanced T1W (CE T1W).

Exclusion criteria are:

1. Patients with post-operative MRI of single BM as initial imaging data;
2. Patients that lacked any MRI investigations;
3. Incomplete MRI investigations (lacking one or more than one sequence detailed in the inclusion criteria).

After the selection of the patients, we followed their evolution for a period of time varying from a minimum of one month to a maximum of 52.1 months. Our end date was 16 June 2024. The survival time was calculated from the histopathological diagnosis of BM till the death date or end of follow-up period of the study.

### 2.2. The MRI Protocol

In order to examine BM features we used a Signa Explorer General Electric Medical Systems MRI machine with a field strength of 1.5 Tesla. With the aim of obtaining maximum information on MRI characteristics, we included sequences such as T1W, CE T1W, T2W, T2W FLAIR, essential for the diagnosis of BMs [4], as well as SWI and DWI/ADC. These sequences are extremely useful in visualizing tumoral behavior, as “blooming” on SWI represents hemorrhage or vascular signal, detecting the hemoglobin inside the blood vessels or in the perivascular space, and high DWI signal with low ADC map values corresponds to high cellular count [25]. As indicated by the EANO-ESMO guidelines we included 3D post-contrast T1W, acquired with a delay of several minutes [4]. The MRI acquisition parameters are listed in Table A1 in the Appendix A section.

### 2.3. Data Collection

We extracted two different sets of data: data at the beginning of the study regarding the patients’ status and metastatic burden, and follow-up data represented by the treatment, post-treatment evolution and OS.

Regarding post-treatment evolution we included post-surgical or radiosurgery complications like distant brain failure after SRS/SRT, local recurrence after surgery or histologically confirmed radio-necrosis after radiotherapy. Other complications taken into consideration were infections or strokes.

Importantly, post-treatment complications were collected to characterize the cohort and clinical course, but they were not used as predictors in the ML models, because they occur after baseline and may be directly influenced by treatment and outcome-related processes.

The therapeutic options that have been used are: surgical resection, radiosurgery, WBRT, and in fewer cases CHT and best supportive care (BSC).

Even though the Karnovsky prognostic score (KPS) was used in deciding which patients should undergo SRT/SRS—KPS > 70 for the 22 patients treated with GK—the score could not be included as a variable in our study due to missing data in the groups of patients with other treatment options.

To describe the metastatic lesions, we analyzed MRI features such as volume, peripheric edema, the presence of central necrosis or cystic components, restricted diffusion, vascularization, internal hemorrhage and localization.

The lesions were grouped by the greater diameter value: BMs less than 5 mm, between 5 and 10 mm, and equal to or above 10 mm. We estimated the volume of the lesions greater than 10 mm using the ellipsoidal method [26]. For the lesions less than 10 mm only one diameter was obtained, and the volume was calculated by applying a spherical volume formula.

This approach is similar to RANO-BM or RECIST 1.1 criteria where lesions are separated in measurable (diameter greater than or equal to 10 mm) and non-measurable (diameter less than 10 mm) [27]. The difference is that we calculated the volume of both measurable and non-measurable BMs, as usually only measurable lesions are further estimated in follow-up MRI investigation, while the non-measurable ones are noted for presence or absence and their number [27].

### 2.4. Statistical Analysis

We organized a dedicated database in Excel in order to categorize patient data across three primary domains: clinical characteristics, MRI-based morphological features, and treatment protocols.

We separately analyzed prognostic factors that depend on the patient characteristics: age; sex; comorbidities (diabetes, smoking status, presence of extracranial metastases, synchronism of BM and primary tumor diagnosis, leptomeningeal metastases, complications); the imaging features of the secondary brain parenchymal lesions (location, type of lesion—solid (with/without central necrosis), cystic or mixed); the presence of diffusion restriction; intralesional hemorrhage; and peritumoral edema.

The volume and number of the BMs, the primary tumors’ nature and treatment options (including gamma knife (GK), surgery, WBRT, CHT and BSC) are additional variables that were examined.

We generated the explanatory data analysis (EDA) for the categorical and numerical variables [28]. We then used the Kaplan–Meier (KM) method to obtain the survival curve for every attribute and the log-rank test to appreciate the difference in survival between groups [29,30].

The univariate Cox proportional hazard was additionally applied for all the significant variables in the univariate survival analysis, calculating the *p*-value, the hazard ratio (HR) and the intervals of confidence (CIs) [31]. Multivariate Cox Regression analysis was also useful in order to compare survival rates between groups with similar covariates (treatment, contrast intake type and volume) [31]. For the survival analysis of metastatic features, we used the Cox Regression function with the intra-patient clustering parameter set.

Treatment variables (surgery, GK/SRS/SRT, WBRT, and CHT) are initiated after diagnosis and are influenced by baseline prognosis (confounding by indication) [32].

In this retrospective dataset, treatment was encoded as to whether a patient received a given modality during their clinical course rather than as a time-dependent exposure. Therefore, Cox model hazard ratios involving treatment indicators may be affected by post-baseline bias (including immortal time bias) and should be interpreted as descriptive associations (treatment-conditional comparisons) rather than causal effects or baseline prognostic factors.

Whenever survival analyses were performed at the lesion level, Cox proportional hazard models were fitted using robust variance estimates clustered by patient identifier (cluster_col), so that statistical inference (standard errors, confidence intervals, and *p*-values) accounts for intra-patient clustering.

For every test, we considered a *p*-value less than or equal to 0.05 statistically significant. Log-log plots of the Cox Regression tests were also generated in order to visually verify the HR assumptions [31].

### 2.5. Data Processing

The analysis started from a curated clinical–radiological spreadsheet, where each patient could contribute multiple rows (one per metastasis). We organized the study database in Excel and subsequently processed and analyzed it in Python version 3.13.5.

Prior to modeling, we performed a structured data-cleaning step: we harmonized categorical labels across the entire cohort, converted all binary variables to a unified 0/1 representation, and verified valid ranges/units for continuous variables.

Missing values were handled using simple, transparent strategies: for continuous/numerical variables, missing values were imputed with the mean calculated on the training set; for binary/categorical variables, missing values were imputed with the most frequent category (mode) from the training set [33].

When volumetry was missing, lesion volume was approximated from recorded size using a sphere-based formula and subsequently binned into clinically interpretable volume categories; additional composite variables (“no treatment”) and patient-level aggregations (metastasis count and summary volume statistics per patient) were created to support analyses at both lesion and patient granularity.

Categorical string variables were encoded using one-hot encoding. Continuous variables were standardized using z-score normalization (StandardScaler) [34].

Diagnosis and death dates were parsed from day–month–year strings and used to derive time-to-event endpoints in months: overall survival for deceased patients (death minus diagnosis) and follow-up time for censored patients computed up to a fixed analysis date [35].

Imaging-derived descriptors were normalized into consistent categorical/binary features (edema, vascularization, hemorrhage, diffusion restriction, contrast pattern, localization, side) [36].

To minimize information leakage, the temporal split was performed at the patient level: patients diagnosed in 2019–2023 were used for training (*n* = 87) and patients diagnosed in 2023–2024 were held out for testing (*n* = 22). This approach corresponds to temporal validation and lies between internal and external validation [37].

### 2.6. AI Algorithms

Four machine learning (ML) algorithms were implemented in evaluating the prediction of survival at 6 months (selected as threshold value) from the time of diagnosis. These models were developed using only processed data, in three different setups: the first used only patient data, the second included only MRI features, and finally the third assessed both sets of data together.

Given the cohort size (*n* = 109 patients; training set *n* = 87), the ML component was designed primarily as an exploratory, comparative experiment to quantify the incremental value of adding metastasis-level MRI information beyond patient-level clinical/treatment variables.

The four machine learning algorithms implemented are:

- Extreme Gradient Boosting (XGBoost) is a machine learning algorithm particularly well-suited for imbalanced datasets, as it can handle classes with significantly different frequencies and provide accurate predictions despite the imbalance [38]; the model’s parameters were *n*_estimators = 50, max_depth = 2, learning_rate = 0.05.
- Neural Networks (NNs) are a machine learning algorithm that uses mathematical representations of neurons (being built similarly to how human brain neurons work) suited for complex feature interactions, considered a deep learning model when more than 2 hidden layers are present; in our implementation we chose a shallow NN—having a single hidden layer, using ‘ReLU’ activation function [39,40,41]. The model’s parameters are detailed in Table 1, with a total of 113 parameters, of which 97 were trainable and 16 non-trainable. We used an ‘Adam’ optimizer with a learning_rate of 0.001, with no regularization method, loss function = ‘binary_crossentropy’. We trained the NN for 50 epochs, with a batch_size of 8.
- K-Nearest Neighbor (KNN) is a classification or regression ML model that predicts the value of data based on the average value calculated from a number of *k* nearby data points (neighbors) [42]; The model’s parameters were n_neighbors = 3, weights = uniform.
- Random Forest (RF) is a largely applied machine learning model that uses multiple decision trees, considered suitable for heterogeneous datasets due to lower overfit predisposition [16,43]. The model’s parameters were *n*_estimators = 100, max_depth = 5.

To reduce overfitting, we constrained model complexity, tuned hyperparameters using 5-fold cross-validation and grid-search procedures within the training set, and reported final performance on a temporally separated, patient-level held-out test set.

### 2.7. Model and Feature Importance Assessment

We compared the classification power of the four different AI prediction models after their training and internal validation by calculating specific metrics [44,45]:

- Accuracy—the capacity of the model to estimate the true positives and true negatives from the total of true and false negatives and positives;
- Precision—the capability to identify the true positives from the total positive results;
- Specificity—identification of the true negatives from the sum of the false positive results and true negatives;
- Recall or sensitivity—demonstrates the model’s sensitivity to detect true positives from the sum of the false negatives and true positives;
- F1-score—the harmonic mean between sensitivity (recall) and precision;
- The time-dependent receiver operating characteristics (ROCs) and the area under the ROC curve (AUC) summarize the discriminatory capability of the model.

We implemented the Shapley Additive Explanations (SHAP) method for every model in the complete dataset to attain feature importance [46]. SHAP assesses the degree of contribution of every attribute by also representing if the variable has a positive or negative effect on the prediction models, thus making it easier to interpret the results [46].

### 2.8. Processing Environment

Python (version 3.13.5) was utilized in order to perform the statistical analysis. The Python packages used were: ‘pandas’, ‘keras’, ‘scikit-learn’, ‘xgboost’, ‘shap’ and ‘matplotlib’.

## 3. Results

### 3.1. Patient Characteristics

Our cohort consisted of a total of 109 patients, 38 (34.9%) women and 71 (65.1%) men, with ages ranging from 21 to 83 years old with a median age of 73 years old.

At the end of the monitoring period, 21 of 109 (19.3%) patients were still alive.

The characteristics of the patients included in this study and their implication in OS are listed in Table A2 and Table A3, found in the Appendix A section.

**Figure 2:**
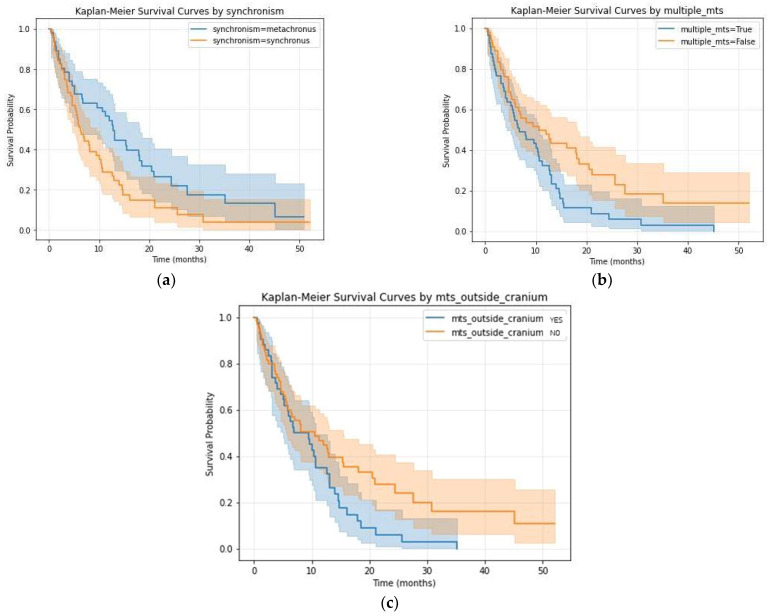
KM survival curves for clinical data: (**a**) metachronous vs. synchronous BMs, *p*-value of 0.02; (**b**) single vs. multiple BMs, *p*-value of 0.01; (**c**) presence of extracranial metastases, *p*-value of 0.04.

The importance of every variable in survival analysis has been estimated using the log-rank test; the KM curves of the ones that were significant (log-rank *p*-value ≤ 0.05) are visible in Figure 2. Consequently, by applying the univariate Cox proportional hazard model, we calculated the HR, *p*-value and CI (Table 2).

The time of BM diagnosis in relation to the diagnosis of the primary tumors had an important implication in survival (*p* = 0.02—log-rank, Figure 2a). Patients with synchronous diagnosis (diagnosis of both BMs and primary tumor at the same time) had a poorer prognosis compared to the patients with a metachronous diagnosis (patients who were already diagnosed with different primary tumors and had been monitored and treated).

By implementing Cox Regression analysis for the synchronous group, we obtained a *p*-value of 0.02, HR = 1.68 and CI (1.09–2.60) suggesting a higher risk (68%) of death compared to the metachronous group. In our cohort, a majority of the patients were diagnosed with stage IV tumoral disease (synchronous—63 patients (57.8%)) and 46 (42.2%) developed BMs after being diagnosed with a primary tumor (metachronous).

The number of secondary lesions for patients varied from 1 (single BM) to 30, with a median number of 2. The number of patients with a single BM was 54 (49.54%) and they had a greater rate of survival compared to the 55 (50.46%) patients who had been diagnosed with multiple secondary brain lesions (*p* = 0.01 by both log-rank and Cox Regression, with HR = 1.73, and CI = 1.13–2.66).

The presence of other systemic metastases at diagnosis has been proven to be important in our dataset, as there was a significant difference in survival (*p* = 0.03—log-rank, *p* = 0.04, HR =0.63, CI (0.41–0.97)—Cox Regression) with the longest survival time being 52 months for patients without systemic tumoral spread compared to 32 months for the other group.

No difference has been found in survival time using the log-rank method regarding the age of the patients (less than 65 years old compared to more than 65 years old, *p*-value of 0.4), the sex of the patients (*p* = 0.1), the type of primary tumor, the presence of diabetes (*p* = 0.47), the smoker status (*p* = 0.41), leptomeningeal involvement (*p* = 0.98) or other post-treatment local complications (*p* = 0.59).

### 3.2. Metastatic MRI Features

In total we analyzed the MRI morphology of 370 secondary brain lesions from the 109 patients included in the study. The characteristics of the secondary brain lesions are listed in Table A4 in the Appendix A section along with the EDA for OS.

Using the univariate and multivariate Cox proportional hazard method, we calculated the importance in the prediction of OS for every variable (Table 3).

**Figure 3:**
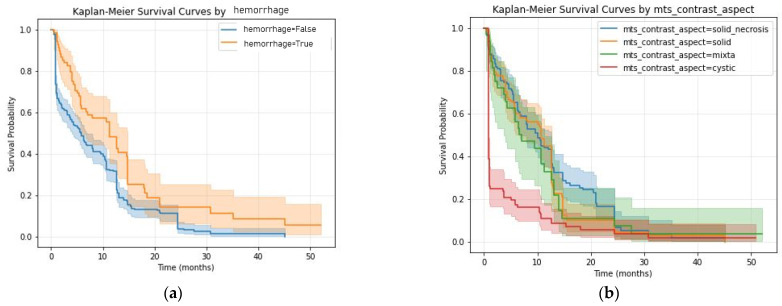
KM survival curves for relevant MRI morphological data: (**a**) hemorrhage; (**b**) contrast intake type.

The KM curves of the significant MRI features are presented in Figure 3.

A surprising result was the *p*-value of 0.02, HR = 0.59 (CI = 0.38–0.92) Cox Regression, for internal bleeding, suggesting tumoral hemorrhage in small quantities (we mostly observed microbleed lesions in the tumoral tissue) might be associated with better outcomes. The 68 (18.38%) lesions with internal hemorrhage showed better survival rates at any time compared with the other 302 metastatic tumors (81.62%) (Figure 3a).

The lesions were classified into solid, with (138—37.3%) or without central necrosis (107—37.3%), cystic (93—25.2%) or mixed lesions (32—8.6%). The solid contrast intake types had a HR < 1 and *p* values of 0.04 (solid) and 0.02 (solid with necrosis) with a better survival rate comparative to cystic and mixed lesions.

Additional morphological features including internal vascularization, peripheric edema, restricted diffusion, volume, anatomical localization (supratentorial vs. infratentorial) and hemispheric laterality (left vs. right) did not reach statistical significance.

The implications of these results are further explained in the Discussion section.

### 3.3. Treatment Options

Regarding the treatment options, they varied due to changes in therapy protocols and availability at our hospital. Patients diagnosed from 2019 to 2022 were mainly surgically treated (99 patients in total—90.8%); the majority of the patients diagnosed from 2022 onwards were also treated with gamma knife (GK) SRT (22 patients—20.2%).

Table A7 (in the Appendix A section) depicts the number of patients treated with the different methods and their OS time in months.

To summarize, 22 of the cases that were surgically treated (99/109) also received GK therapy. The SRS/SRT protocols differed as follows:

- 10 received adjuvant SRT;
- Another five patients previously underwent adjuvant WBRT (here SRT was used as the secondary treatment after relapse);
- In three cases, GK was performed both before and after the surgery;
- There were four cases where only neoadjuvant GK was performed, with the administration of one radiotherapy session one day before surgery.

The most used adjuvant SRT protocol consisted of the administration of 30 Gy in three different fractions of 10 Gy at an interval of two weeks, beginning 1 month after surgical intervention.

Of the rest of the WBRT-treated patients, three underwent radiotherapy after the surgical resection and only one was treated by WBRT alone. In total, nine patients were treated with WBRT. CHT was an option for only 13 patients that benefitted from other therapies as well. Only six patients were followed-up with BSC and symptomatic therapy management.

**Figure 4:**
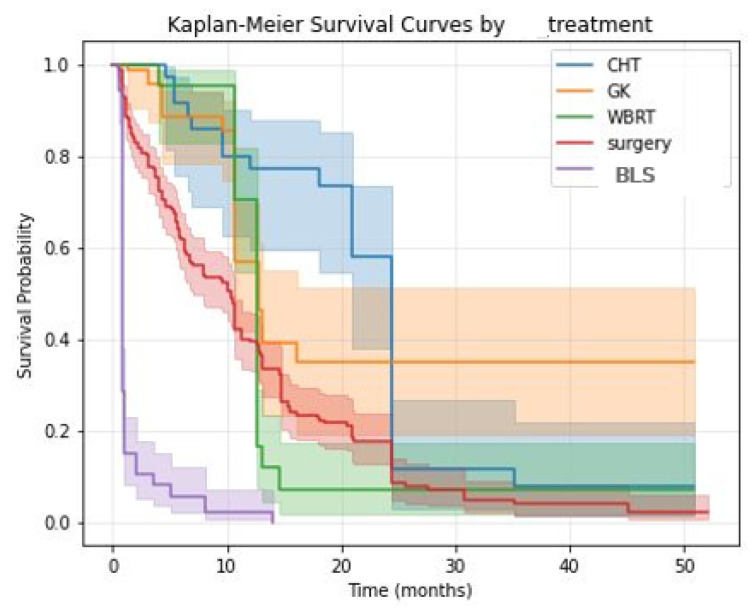
KM survival curves for treatment options compared to BSC and log-rank *p*-values: CHT *p*-value of 0.05; surgery *p*-value of 0.01; GK *p*-value < 0.005; WBRT *p*-value of 0.03.

In this observational cohort, active treatment was associated with longer OS compared to BSC, as shown in Figure 4, with log-rank *p*-values of 0.05 for CHT, 0.01 for surgery, 0.03 for WBRT and <0.005 for GK. Both in the univariate and multivariate Cox Regression analyses (except CHT with a univariate *p*-value of 0.055), treatment indicators were associated with HR < 1 compared to BSC (Table 4).

However, because treatments are initiated post-diagnosis and are influenced by baseline risk, these estimates should be interpreted as treatment-conditional associations rather than causal effects.

### 3.4. Prediction Performance of AI Models

For the training of the four ML models, we used 80% of our data, selecting the patients from 2019 till the end of 2023 (87 patients in total—79.8%). For the temporary validation, the other 22 patients (20.2%) diagnosed from 2023 to 2024 were used as new data.

We chose as variables the first ten features that obtained the lowest *p*-value in the log-rank and Cox Regression analysis. For the clinical dataset we used these variables: ‘synchronism’, the number of lesions, the presence of systemic secondary lesions and the treatment (‘CHT’, ‘surgery’, ‘WBRT’, ‘GK’ and ‘BSC’). The MRI morphological dataset was composed of the presence of ‘vascularization’, ‘hemorrhage’, ‘peripheric edema’, ‘restricted diffusion’, the type of contrast intake and the volume of lesions expressed in cm^3^. These are all the features used in the models’ input.

Regarding the number and volume of lesions, we opted to include numerical values as opposed to categorical ones, in order to appreciate the continuity of the data and its effect on survival.

Because treatment variables are decided after diagnosis, the models should be interpreted as treatment-conditional survival prediction models rather than purely baseline prognostic ones. Therefore, we avoid causal claims from these predictors and present them as part of an exploratory, real-world prediction setting.

**Figure A1:**
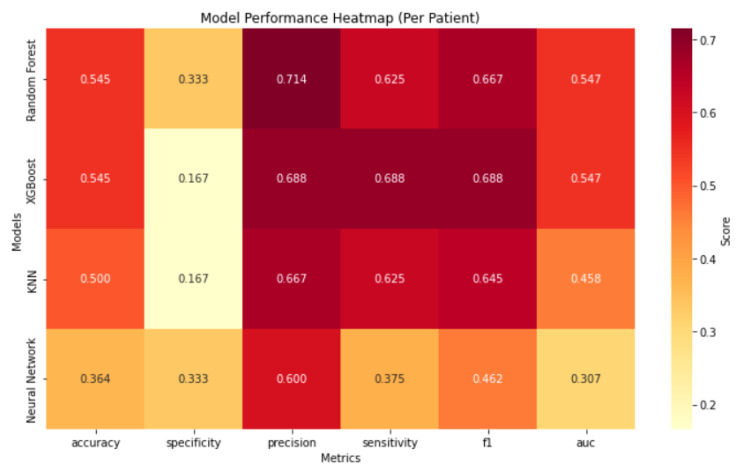
Heatmap showing prediction performance using only clinical data.

**Figure A2:**
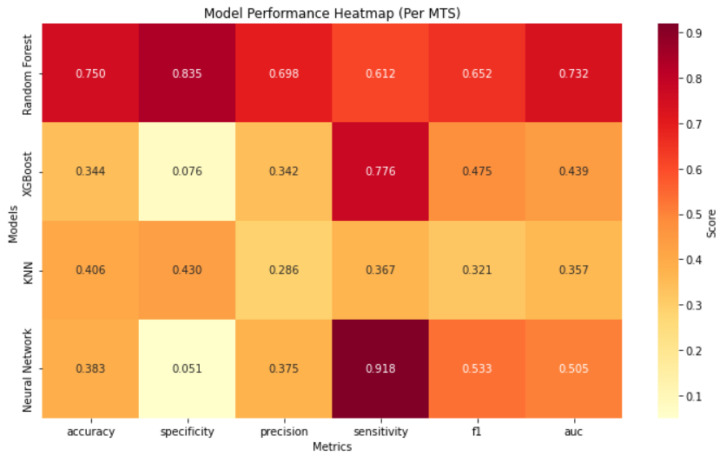
Heatmap showing prediction performance using only MRI morphological data.

**Figure A3:**
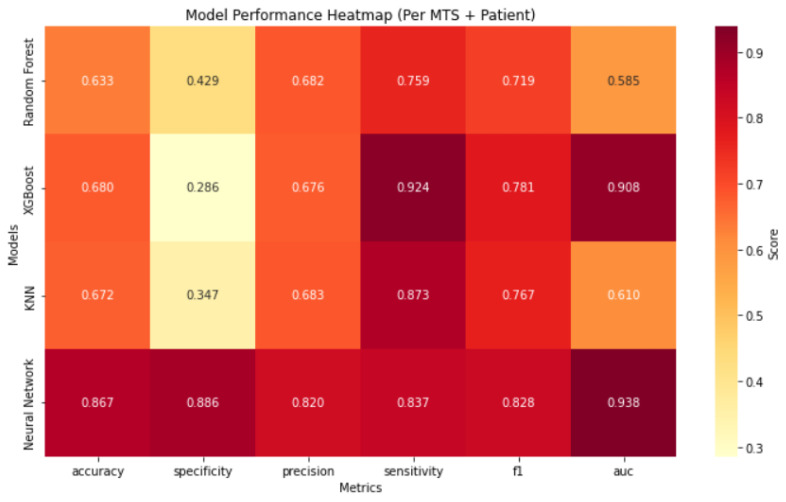
Heatmap showing prediction performance using clinical and MRI morphological data.

We analyzed the performance parameters of the prediction models, first by only including clinical data (Figure A1, found in Appendix B), secondly with only MRI morphological data (Figure A2, found in Appendix B) and, finally, both sets of data at once, visible in the heatmap Figure A3, found in Appendix B.

With regard to the clinical data (Figure A1), the models performed the poorest with low accuracy (54% for XGBoost, 54% for RF, 50% KNN and 36% NN), very low specificity (16% XGBoost, 33% RF, 16% KNN and 33% for NN); slightly better precision (68% XGBoost, 71% RF, 66% KNN and 60% NN); low sensitivity (recall) (68% XGBoost, 62% RF, 62% KNN and 37% NN), low F1-score (68% XGBoost, 66% RF, 64% KNN and 46% NN), and poor AUC (0.54 XGBoost (CI = 0.26–0.81), 0.54 RF (CI = 0.23–0.83), 0.45 KNN (CI = 0.25–0.68) and 0.30 NN (CI = 0.11–0.65)). The overall best model in this scenario was XGBoost, but that had a poor discriminatory power and very low specificity.

In the MRI features dataset (Figure A2), the models performed similarly to the first case. RF was the best prediction model with low accuracy (75%); moderate specificity (83%); poor precision (69%), recall (61%), and F1-score (65%); and an AUC of 0.73 (CI = 0.63–0.83). The other models obtained lower values, except for sensitivity (recall), with a value of 77% for the XGBoost model and 91% for the NN model.

**Figure 5:**
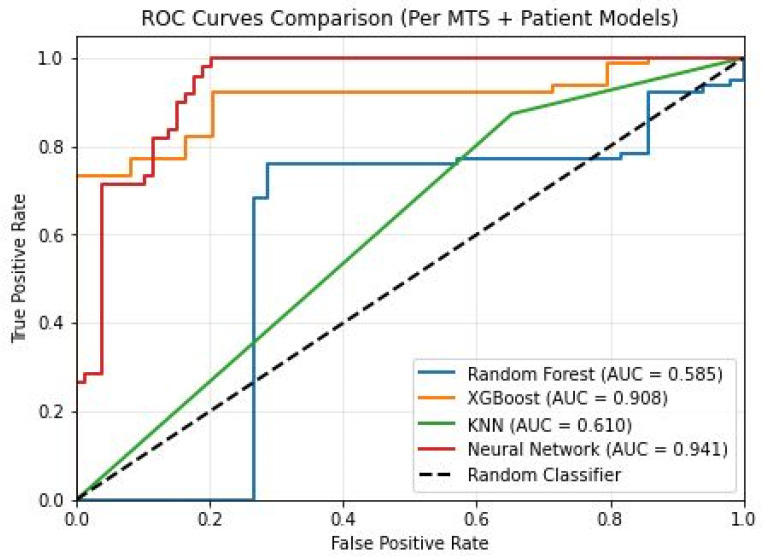
AUC for every model after training on both clinical and morphological data.

After training the ML models on both sets of data, we obtained different results regarding the models’ classification power; the AUC values achieved by every program are presented in Figure 5.

Models that displayed greater accuracy than the Random Classifier are NN and XGBoost, with an excellent discriminatory power (AUC) of 0.93 (CI = 0.89–0.97) and 0.90 (CI = 0.85–0.95) respectively. The other two models, KNN and RF, had a poor discriminatory power with an AUC of 0.61 (CI = 0.53–0.68) and 0.58 (CI = 0.47–0.69) respectively.

In Figure A3 in Appendix B, the other metrics are also displayed.

Shallow NN proved to be the most performant model of the four, with an accuracy of 86%, precision of 82%, specificity of 88%, recall of 83%, an F1-score of 82% and an AUC of 0.93 (CI = 0.89–0.97).

XGBoost follows the shallow NN model with an accuracy of 68%, precision of 67%, specificity of 28%, recall of 92%, an F1-score of 78% and an AUC of 0.90 (CI = 0.85–0.95). KNN was the model that obtained the next best results, with an accuracy of 67%, precision of 68%, specificity of 34%, recall of 87%, an F1-score of 76% and an AUC of 0.61 (CI = 0.53–0.68) with lower discrimination.

The least performant model was RF, displaying the lowest metrics: accuracy of 63%, precision of 68%, specificity of 42%, recall of 75%, an F1-score of 71% and an AUC of 0.58 (CI = 0.47–0.69) with poor discrimination.

### 3.5. Model Interpretability

**Figure 6:**
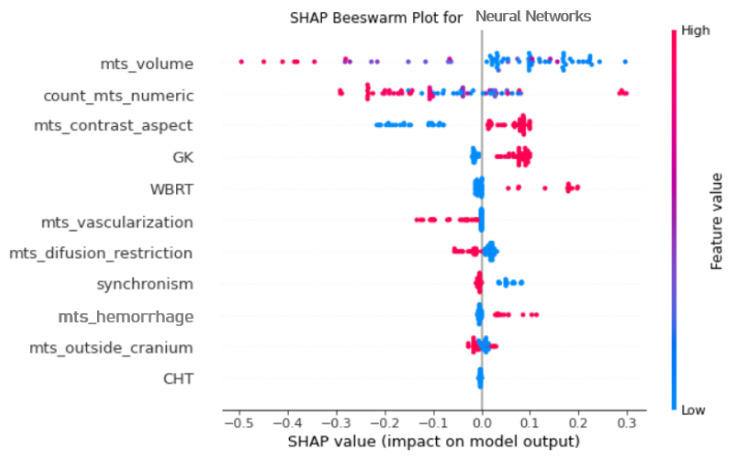
SHAP showing feature relevance using clinical and MRI morphological data for the NN model.

With the aim of identifying the most important MRI features and their implication in the models’ results, we implemented the SHAP analysis. As NNs were the most performant algorithm in the last case (all metrics included) we opted to show the feature importance for the shallow NN model (Figure 6).

As shown in the SHAP heatmap, the volume of lesions has the greatest impact on the model performance as follows: greater volumes are associated with poor estimated prognosis. The number of lesions is also associated with a negative impact on the predicted survival, as numerous BMs mostly correspond to negative values on the impact axis, with a cluster of numerous lesions associated with a positive impact (seen in Figure 4 as numerous bright pink dots—associated with an outlier).

Table A5 and Table A6 in Appendix A show the number, diameters (mm) and volumes (cm^3^) of the BMs.

Another morphological feature associated with a positive effect on the predicted OS is the presence of a solid portion of the lesion that has contrast intake (pink dots), as opposed to cystic or mixed lesions (blue dots) that are associated with poor estimated survival.

Other MRI features found to be valuable in the model prediction are the presence of internal vascularization and restricted diffusion, which are associated with a poor prognosis. Internal hemorrhage appears to have a positive impact on the estimated OS.

Even though the presence of extracerebral metastases and synchronism had been selected by SHAP in the NN model, their implication in survival prediction is low, as both are really close to the zero value on the impact axis.

Concerning treatment methods, WBRT and GK are greatly associated with positive values, thus having a positive impact on the experimental prediction model.

## 4. Discussion

### 4.1. Log-Rank and Cox Regression Results’ Implications

Regarding clinical characteristics, only a limited number of variables emerged as significant prognostic indicators for survival: the presence of extracerebral metastases, the synchronous presentation and multiple brain lesions. The high number of BMs and the presence of extracerebral metastases are poor prognostic factors that are already used in different clinical settings, being included in prognostic assessment tools, like GPA [9].

The chronological interval between the primary tumor diagnosis and the detection of brain involvement was identified as a critical determinant of OS.

In the case of a synchronous diagnosis of the primary tumor and the BMs, the local development and systemic spread of cancer have already taken place, as the involvement of brain parenchyma occurs later in extracranial neoplasia. Meanwhile, in the case of a metachronous diagnosis of BMS, the patient is known to suffer from a primary tumor. Treatment of the primary tumor and screening for the onset of BM benefits the patients and ensures a longer OS [1]. Other studies reached similar conclusions and sustained the theory that the time of diagnosis might be an important prognosis factor [47]. However, synchronous diagnosis should not be the reason to avoid performing the surgical resection of the BMs, as Potthoff et al. found that there was no difference in survival after surgical removal compared with the metachronous group [48].

Although the nature of the primary tumors has been assessed, none was specifically more aggressive than others in our cohort of patients. Only melanoma metastases showed a poor but not significant effect on survival (*p* = 0.07, log-rank). Melanoma patients had the shortest OS, with a median survival of 4 months, in agreement with other findings [1].

Including the MRI features gave our work more robustness in survival prediction. Nonetheless, the focus on the MRI aspects of BMs is a novel approach, as the majority of the studies conducted prior used clinical data only [47].

Of the variables that we included, the type of contrast intake and the presence of internal hemorrhage were the most important features.

The presence of hemorrhage could be the result of a visible blood–brain barrier rupture (microhemorrhages) that ensured a better response to local or systemic treatment. Another theory could be that the blooming on SWI has been wrongly assessed as being of a vascular nature instead of being calcification. This could be avoided by introducing a native computer tomography examination for the same patients, prior to treatment, as the values in Hounsfield units for calcification are considerably higher than the ones suggesting hemorrhage [49]. Another reason for the relevancy of this feature could be the residual confounding resulting from the statistical analysis.

Compared to the other contrast intake types, the cystic and mixed lesions had poorer prognoses. Cystic lesions are also known to be more resistant to radiotherapy [2]. This resides in the fact that in a fluid-filled lesion, SRT can only be effective on the walls of the lesion as the cyst can contain cellular debrides and other residues, but not viable tumoral cells. In one study that assessed morphological MRI and volumetric data, the thickness of the rim was predictive of better progression-free survival, after radiotherapy [6].

### 4.2. ML Model Results’ Implications

Using the same four models for three different situations enabled us to compare them not only by specific metrics but also depending on the data that was submitted.

When both sets of data, containing clinical and treatment protocol information, were evaluated together with MRI features, every model had a better performance status than when analyzing the sets of data separately.

The analysis of only clinical data obtained the worst outcomes and the models were the least performant in OS prediction, the best one being RF (see Figure A1 in Appendix B).

The assessment of only MRI features allowed the models to achieve better results, but they were still not competitive enough (see Figure A2 in Appendix B).

The addition of MRI features to the clinical ones has been proven to boost the prediction of OS by the ML models, with greater metric values and better discrimination (see Figure A3 in Appendix B). In this case, NNs was the model that obtained the greatest prediction values and discriminatory power (AUC = 0.93).

Regarding feature importance, the SHAP analysis easily identified the more relevant variables and their implication in the models’ capability to estimate OS (see Figure 4).

The clinical features that were selected are the number of lesions and presence of systemic metastases, in correlation with the survival analysis. These two factors are already part of different prognostic appreciation and risk stratification scales [3]. Synchronism was also selected with a poor prognostic value. In some studies, synchronous diagnosis in younger patients is correlated with longer survival compared to metachronous diagnosis in patients over 65 years of age [1].

Other clinical characteristics did not reach significance: age, sex, diabetes and smoker status. Also, the presence of leptomeningeal involvement did not affect survival, in contradiction with other findings, as leptomeningeal metastases are known to be an independently poor prognosis factor [50,51].

The MRI features that obtained importance are: the volume of lesions, contrast intake type (solid with/without central necrosis versus cystic and mixed), vascularization, hemorrhage and restricted diffusion.

Recent studies consider the total volume of BMs to be more important in OS prediction than the number of lesions [52]. In the case of SRT, for example, the total volume of lesions can be more relevant than the number because the permitted radiation dose is inversely related to the total volume of the lesions [2,53]. Other studies found that the median volume and size of the lesions are important prognostic factors in BMs from different primary tumors [16,54].

The NN and RF models found that restricted diffusion is associated with poor prognosis. As previously stated, the presence of restricted diffusion in any brain tumor is associated with worse outcomes; by its nature, high tumoral cellularity is linked with an aggressive behavior and poor OS [18]. To an extent, restricted diffusion in BMs is considered a poor prognosis factor. Other studies comparing different types of BMs found that restricted diffusion can differentiate SCLC metastases from NSCLC, as SCLC showed greater cellularity and worse prognosis, despite being more radiosensitive [55,56].

All the models found that the presence of a cystic component in the cystic and mixed types of BM was associated with poor prognosis compared to solid lesions [2].

The presence of internal vascularization was also found to be of importance, having a negative impact on OS estimation by the NN and RF models. Contrary to vascularization, the presence of microhemorrhages was found to be positively correlated with survival by the NN model. This finding is in contradiction with general knowledge regarding internal tumoral bleeding, which is often correlated to hemorrhagic BMs with poor prognosis (melanoma) [1].

Possible reasons for such results have been previously explained. Additionally, because metastatic MRI features are less explored compared to primary brain tumors, there is insufficient data to make pertinent conclusions, and also a limited number of studies to compare our results with. Thus, the need to further explore how internal hemorrhage and blood–brain barrier integrity could affect local treatment outcomes remains of great importance.

All the ML algorithms performed better in predicting OS at 6 months when MRI features were added to clinical data. These findings once again emphasize the need to develop models that include the morphological MRI characteristics of BMs that can further contribute to clinical decision-making.

### 4.3. Limitations

Because all the patients included in this study were admitted upon diagnosis in a neurosurgery hospital, all of them had different neurological symptoms. This can be viewed as a selection bias, as no asymptomatic patients were included in the study.

Likewise, the fact that we included only histopathologically confirmed patients can also be seen as a selection bias, as multiple patients were excluded from our study; however, this was a critical step in order to ensure the quality of our data and also the possibility to evaluate the prognostic value of the nature of the primary tumor.

Because of the retrospective nature of the study, valuable data such as immunohistochemical subtypes, the differentiation grade and the expression of specific receptors was missing, thus limiting us in making any correlation with specific primary tumors’ molecular subtypes and OS.

Similarly, the KPS was missing. This is an important limitation because omission of the KPS may reduce comparability with prior prognostic models. Its prognostic value has long been demonstrated by numerous studies and it is currently used, alone or in prognostic scales, such as GPA, for the stratification of patient risk and the determination of the treatment option.

There are, however, studies which did not find a correlation between the GPA score and OS [6]. Other studies found that GPA had a limited precision for prognostic estimation of only 3–6 months [8]. Considering that the OS of metastatic patients is getting longer because of the improvement in treatment protocols [57], new and more comprehensive prognostic scores should be developed to be used over a longer period of time.

Although the quality of the data is satisfactory, the number of patients (109) and secondary lesions (370) could also poorly affect the ML models, leading to model instability, as they are known to operate on more data. The temporal validation of the four models is another limitation of the study, as external validation is usually superior.

Our research does not seek to claim definitive development and validation of a generalizable machine learning model. Future work can include multicenter cohorts for confirmatory modeling consistent with external validation.

## 5. Conclusions

Our results confirm that including the morphological MRI features of BMs in the estimation of OS significantly contributes to the enhancement of ML algorithms’ prediction and discriminatory capacity.

The presence of extracranial metastases, synchronism, a higher number of lesions (clinical variables), cystic enhancement and internal vascularization (morphological variables) were selected by the Cox Regression test as significant variables (*p* < 0.05) in defining a poor prognosis.

The SHAP analysis, used in the importance assessment of variables in the ML models, additionally identified volume (greater values), the presence of restricted diffusion and the cystic component as significant features.

This study could contribute to selecting important MRI metastatic features to be included in future research regarding survival and prognostic appreciation, with the aim to help physicians in selecting an appropriate treatment protocol and to ensure a personalized approach in BM management.

## Figures and Tables

**Table 1:** Parameters for the shallow NN model.

Layer	Output Shape	Parameters
Dense	(None, 8)	72
Batch normalization	(None, 8)	32
Dropout	(None, 8)	0
Dense	(None, 1)	9

**Table 2:** Univariate Cox Regression results for clinical data.

Data	HR	CI (−95%)	CI (95%)	*p*-Value
Metachronous (42.2%)	Reference	Reference	Reference	Reference
Synchronous (57.8%)	1.68	1.09	2.60	0.02
Single metastasis (49.54%)	Reference	Reference	Reference	Reference
Multiple metastases (50.46%)	1.73	1.13	2.66	0.01
EM * (38.53%)	Reference	Reference	Reference	Reference
No EM * (61.47%)	0.63	0.41	0.97	0.04

* Extracranial metastases.

**Table 3:** Univariate and multivariate Cox Regression results for MRI features.

Feature	HR	CI (−95%)	CI (95%)	*p*-Value
No vascularization (74.6%)	Reference	Reference	Reference	Reference
Vascularization (25.6%)	0.76	0.45	1.15	0.17
No hemorrhage (81.62%)	Reference	Reference	Reference	Reference
Hemorrhage (18.28%)	0.59	0.38	0.92	0.02
No edema (43.51%)	Reference	Reference	Reference	Reference
Edema (56.48%)	0.53	0.26	1.07	0.07
No diffusion (48.38%)	Reference	Reference	Reference	Reference
Diffusion (51.62%)	1.59	0.85	2.95	0.13
Cystic (25.2%)	Reference	Reference	Reference	Reference
Mixed (8.6%)	0.39	0.14	1.09	0.07
Solid (28.9%)	0.37	0.14	0.97	0.04
Solid with necrosis (37.3%)	0.33	0.12	0.87	0.02
Diameter < 5 (72.5%)	Reference	Reference	Reference	Reference
≥5 and <10 (6.5%)	0.63	0.39	1.00	0.053
≥10 (21%)	0.70	0.39	1.27	0.25

**Table 4:** Univariate and multivariate Cox Regression results for treatment options.

Treatment	Univariate				Multivariate			
	HR	CI (−95%)	CI (95%)	*p*-Value	HR	CI (−95%)	CI (95%)	*p*-Value
BSC (6)	Reference	Reference	Reference	Reference	Reference	Reference	Reference	Reference
CHT (13)	0.52	0.27	1.02	0.055	0.37	0.24	0.57	4 × 10^−6^
Surgery (99)	0.4	0.2	0.78	0.01	0.2	0.32	0.73	5 × 10^−26^
SRT (GK) (22)	0.31	0.16	0.63	<0.005	0.48	0.16	0.36	5 × 10^−4^
WBRT (9)	0.37	0.15	0.91	0.03	0.25	0.15	0.27	3 × 10^−12^

## Data Availability

Our data is not publicly available due to ethical restrictions, but it can be obtained from the corresponding authors on reasonable request.

## Abbreviations

The following abbreviations are used in this manuscript:

BM(s)	Brain Metastasis
OS	Overall Survival
WBRT	Whole-Brain Radiotherapy
SRS	Stereotactic Radiosurgery
SRT	Fractionated Radiotherapy
CHT	Chemotherapy
MRI	Magnetic Resonance Imaging
T1W	T1-weighted
T2W	T2-weighted
T2W FLAIR	T2-weighted Fluid Attenuated Inversion Recovery
GPA	Graded Prognostic Assessment
DS-GPA	Disease Specific GPA
RANO	Response Assessment in Neuro-Oncology
RECIST	Response Evaluation Criteria in Solid Tumors
AI	Artificial Intelligence
SWI	Susceptibility Weighted Imaging
DWI	Diffusion-weighted Imaging
ADC	Apparent Diffusion Coefficient
CE T1W	Contrast-enhanced T1-weighted
BSC	Best Supportive Care
GK	Gamma-knife
(KPS)	Karnovsky prognostic score
EDA	Explanatory Data Analysis
KM	Kaplan–Meier
HR	Hazard Ratio
CI	Intervals Of Confidence
ML	Machine Learning
XGBoost	Extreme Gradient Boosting
NN	Neural Network
KNN	K-Nearest Neighbor
RF	Random Forest
ROC	Receiver Operating Characteristics
AUC	Area Under the ROC Curve
SHAP	Shapley Additive Explanations

## Appendix A

The MRI acquisition parameters differ for every sequence (see Table A1); the echo number is equal to 1, and the matrix is 256/256 for DWI/ADC and 512/512 for the other sequences.

**Table A1:** Parameter acquisition for every MRI sequence.

Sequence	Slice Thickness (mm)	Spacing (mm)	TE * (s)	TR ** (s)
T1W	1.8–2	0.8–1	3.5–4.2	8–9.6
T2W	4–5	5	104–110	4101–6215
T2W FLAIR	1.2–1.8	0.6–0.8	119–300	4800–7002
DWI	4–5	4–6	83.59–84.5	4600–8201
ADC	4–5	4–6	83.59–84.5	4600–8201
SWI	3	1–1.5	~49	52–79
CE T1W	1–1.8	0.8–1	3.5–4.2	8–9.2

* Time to echo; ** repetition time.

The characteristics of our dataset are visible in Table A2, where we can find the age at diagnosis, sex, nature of primary tumor, synchronism, number of lesions and other comorbidities. Note that age at diagnosis was further analyzed as numerical data in Table A3.

**Table A2:** Patients’ characteristics and survival time in months (EDA).

Characteristic	Number/Type	Mean Time	SD *	Min	25%	50% (Median)	75%	Max
Age at diagnosis	>65 y.o. (41—59.6%)	9.86	10.04	0.46	3.53	6.46	12.83	50.9
	<65 y.o. (68—62.4%)	11.26	10.36	0.5	3.99	8.8	15.56	52.1
Sex	M (71—65.1%)	10.12	10.76	0.46	3.1	6.7	13.06	52.1
	F (38—34.9%)	11.83	9.15	0.5	4.18	11.31	17.39	35.53
Primary tumor	NSCLC (55—50.46%)	11.10	10.22	0.46	4.46	8.1	14.38	50.9
	SCLC (7—6.43%)	6.24	6.39	0.6	0.88	3.73	11.01	15.56
	Breast (14—12.84%)	16.21	11.02	0.96	7.91	14.11	22.57	35.53
	Digestive (7—6.43%)	8.26	5.91	1.43	4.31	7	11.1	18.6
	Melanoma (12—11%)	6.54	7.26	1.36	1.88	4.58	7	27.6
	Other (10—9.17%)	12.63	14.88	1.83	4.21	6.71	14.06	52.1
	Unknown (4—3.67%)	6.69	5.66	0.5	2.87	6.58	10.4	13.1
Synchronous Metachronous	63 (57.8%)	8.45	8.38	0.5	3.31	6.2	10.41	52.1
	46 (42.2%)	13.87	11.67	0.46	4.23	12.43	20.05	50.9
Number of metastases	Single (54—49.54%)	12.77	11.7	0.46	4.6	8.8	18.45	52.1
	Multiple (55—50.46%)	8.75	8.14	0.5	3.1	6.46	12.48	45.16
LM **	Yes (31—28.44%)	11.07	11.45	0.46	2.51	7.9	14.3	52.1
	No (78—71.56%)	10.6	9.76	0.83	3.77	7.53	14.01	50.9
Extracranial metastases	Yes (42—38.53%)	9.2	7.29	0.5	3.25	7.43	12.96	35.1
	No (67—61.47%)	12	11.67	0.46	4.1	8.03	15.56	52.1
Diabetes	Yes (17—15.6%)	8.91	8.85	0.93	3.53	7	10.66	35.53
	No (92—84.4%)	11.07	10.46	0.46	3.65	8.08	14.9	52.1
Smoker status (declaratively)	Smoker (13—12%)	10.9	11.18	0.60	5.13	9.7	13.03	45.16
	Non-smoker (96—88%)	10.72	10.14	0.46	3.42	7.05	14.9	52.1
Complications	Yes (29—26.6%)	10.73	11.66	0.46	3.03	5.26	14.76	50.9
	No (80—73.4%)	10.74	9.72	0.5	3.95	8	14	52.1

* Standard deviation, ** Leptomeningeal metastasis.

**Table A3:** Patients’ age (numerical values) and survival time in months (EDA).

Age at Diagnosis	Number	Mean Time	SD *	Min	25%	50%	75%	Max
<40	5 (4.6%)	13.52	10.26	1.5	6.56	12.93	19.03	27.6
40–50	12 (11%)	8.83	6.47	1.43	3.74	8.78	12.39	21
50–60	29 (26.5%)	11.82	11.52	0.5	3.1	10.36	17.86	52.1
60–70	52 (48%)	10.98	10.81	0.46	3.65	7.5	13.35	50.9
70–80	10 (9%)	8.24	6.84	0.86	4.35	6.73	9.85	23.4
>80	1 (0.9%)	0.6	-	0.6	0.6	0.6	0.6	0.6

* Standard deviation.

For the MRI features of secondary lesions, an EDA was also performed, visible in Table A4. In this case survival was appreciated taking into regard the imaging characteristics of every secondary lesion for every patient.

**Table A4:** MRI features analyzed for every BM and survival in months (EDA).

MRI Features	Number/Type	Mean Time	SD *	Min	25%	50%	75%	Max
Internal vascularization	Yes (94—25.4%)	9.27	8.68	0.83	2.68	7.05	13.1	52.1
	No (276—74.6%)	7.95	8.19	0.46	0.86	5.86	12.39	50.9
Internal hemorrhage	Yes (68—18.38%)	11.56	10.81	0.6	4.27	9.7	14.76	52.1
	No (302—81.62%)	7.55	7.48	0.46	0.86	5.76	12.3	45.16
Contrast intake type	Solid (107—28.9%)	9.44	7.54	0.5	4.03	10.1	12.66	45.16
	Solid with necrosis (138—37.3%)	10.08	7.97	0.5	3.83	8.08	12.98	45.16
	Cystic (93—25.2%)	3.91	7.55	0.83	0.83	0.83	1.1	50.9
	Mixt (32—8.6%)	9.43	10.16	0.46	2.44	6.78	12.77	52.1
Edema	Yes (209—56.48%)	10.27	9.56	0.46	3.03	7.96	14.13	52.1
	No (161—43.52%)	5.72	5.41	0.5	0.83	4.03	10.36	22.96
Diffusion restriction	Yes (191—51.62%)	6.70	8.67	0.46	0.83	3.1	9.9	52.1
	No (179—48.38%)	9.98	7.61	0.5	4.03	10.1	12.66	45.16
Greater diameter	<5 mm (268—72.5%)	7.67	7.74	0.5	0.86	5.86	12.3	45.16
	Between 5 and 10 mm (24—6.5%)	9.65	7.26	0.6	3.51	7.11	14.96	23.96
	>10 mm (78—21%)	9.99	10.18	0.46	3.05	6.98	13.08	52.1
Site	Supratentorial (275—74.3%)	8.47	8.63	0.46	0.93	6.46	12.66	52.1
	Infratentorial (95—25.7%)	7.77	7.41	0.5	1.83	5.53	10.96	35.53

* Standard deviation.

Because the volume of the BMs turns out to be very important in OS estimation, the following tables, Table A5 and Table A6, contain supplementary information about relations between size (diameter and volume) and the number of secondary lesions.

**Table A5:** Diameters (mm) and volumes (cm^3^) of BMs (EDA).

Greater Diameter of BMs (mm)	BMs	Mean Volume	SD *	Min Volume	25%	50%	75%	Max Volume
<5	268 (72.5%)	0.75	1.08	0.03	0.05	0.2	0.9	4.92
≥5 and <10	24 (6.5%)	7.23	1.61	5.04	5.61	6.82	8.74	9.4
≥10	78 (21%)	31.7	21.55	10.13	16.9	26.03	37.01	121.23

* Standard deviation.

**Table A6:** Total number of BMs per patient and volumes (cm^3^) of BMs (EDA).

Total Number of BMs	Patients	Mean Volume	SD *	Min Volume	25%	50%	75%	Max Volume
1	54 (49.5%)	27.66	25.89	1.67	8.78	19.47	36.99	121.23
1–5	42 (38.5%)	6.9	5.91	0.03	1.77	5.87	10.85	25.58
>5	13 (12%)	3.43	2.12	0.70	1.91	3.42	5.13	7.65

* Standard deviation.

The last table in Appendix A is dedicated to treatment protocols used in our cohort and their implication in OS. Table A7 delivers an overview of treatment choice over survival.

**Table A7:** Treatment options and their OS (months).

Treatment	Number/Type	Mean Time	SD *	Min	25%	50%	75%	Max
Chemotherapy	Yes (13—12%)	18.65	14.03	4.63	6.96	12.3	24.43	50.9
	No (96—88%)	9.67	9.11	0.46	3.09	6.85	13.24	52.1
Surgery	Yes (99—90.8%)	11.27	10.47	0.46	3.88	8.06	15.03	52.1
	No (10—9.1%)	5.42	4.86	0.6	1.21	4.3	7.66	13.96
SRT (GK)	Yes (22—20.2%)	14.44	10.9	1.36	9.65	12.53	15.96	50.9
	No (87—79.8%)	9.8	9.88	0.46	3.05	6.26	13.35	52.1
WBRT	Yes (9—8.3%)	19.45	14.69	4.1	12.06	13.1	21.4	50.9
	No (100—91.7%)	9.95	9.43	0.46	3.1	6.83	13.74	51.1

* Standard deviation.

## Appendix B

In Appendix B we included two heatmaps representing the metric of the ML models used in the appreciation of prognosis at 6 months for BM patients, in the case of using only clinical data (Figure A1), in the case of using only morphological MRI data (Figure A2) and for both sets of data (Figure A3).
